# Validity of smartphone-based retinal photography (PEEK-retina) compared to the standard ophthalmic fundus camera in diagnosing diabetic retinopathy in Uganda: A cross-sectional study

**DOI:** 10.1371/journal.pone.0273633

**Published:** 2022-09-06

**Authors:** Ahmed Mohamud Yusuf, Rebecca Claire Lusobya, John Mukisa, Charles Batte, Damalie Nakanjako, Otiti Juliet-Sengeri

**Affiliations:** 1 Department of Ophthalmology, School of Medicine, College of Health Sciences, Makerere University, Kampala, Uganda; 2 Department of Immunology and Molecular Biology, School of Biomedical Sciences, College of Health Sciences, Makerere University, Kampala, Uganda; 3 Department of Medicine, Lung Institute, College of Health Sciences, Makerere University, Kampala, Uganda; 4 Department of Medicine, School of Medicine, College of Health Sciences, Makerere University, Kampala, Uganda; University of Warmia, POLAND

## Abstract

**Introduction:**

Diabetic retinopathy (DR) is one of the major complications of diabetes mellitus and is a significant cause of blindness worldwide. In Uganda, the prevalence of diabetes is approximately 2.7% of the urban population and 1% in rural areas. Many diabetics cannot access an eye exam due to the lack of less costly and user-friendly equipment that primary eye workers can use. Smartphone-based fundus photography allows for a cheap and mobile fundus examination. The study aimed to determine the sensitivity and specificity of the Portable Eye Examination Kit (PEEK) retina compared to a standard ophthalmic fundus camera (Zeiss Visucam 200) for the diagnosis of DR.

**Methods:**

From January-March 2020, 286 people with diabetes (type 1 & 2) patients were seen at Kiruddu National referral hospital diabetes clinic. All participants had funduscopy with PEEK retina and the standard ophthalmic fundus camera following ophthalmic examination and pupillary dilation. The PEEK retina’s sensitivity, specificity and reliability were determined using an ophthalmic fundus camera as the gold standard.

**Results:**

The participants’ mean age was 51 with a standard deviation of ±11years, 213 (74.5%) were females, and the majority (93.4%) had Type 2 diabetes. The overall Sensitivity of PEEK retina for DR was 84% (95% CI 70.9–83.5), while the specificity was 79.9% (95% CI 76–83.5) with a positive predictive value (PPV) of 30.9% (95% CI 23.2–39.4) and a negative predictive value (NPV) of 97.9% (95% CI 95.9–99.1).

**Conclusions:**

PEEK retina has high sensitivity and specificity, making it suitable for screening and diagnostic purposes. Therefore, we recommend the integration of the PEEK retina in the screening and diagnosis of DR in resource-limited settings.

## Introduction

Diabetic retinopathy (DR), a complication of diabetes mellitus, is a significant cause of avoidable blindness worldwide [1]. Its prevalence is expected to rise since the global prevalence of diabetes mellitus has been trending from 4.7% in 1980, 6.4% in 2010, 8.5% in 2014, to 8.8% in 2015 [2, 3]. In addition, diabetes was the fifth leading cause of death globally, with a prevalence of 463 million people in 2019 [4]. This prevalence is rapidly increasing in Africa and is even faster, with an expected 69% increase in low-income and middle-income countries (LMICs) like Uganda than in high-income countries with a 20% increase [5–7]. In Uganda, the overall prevalence of diabetes was 2.8% in 2016, 2.7% in the urban population and 1% in the rural population. However, there has been no documented prevalence in recent years [8].

With the advance in age and increased therapeutics, there has been an increase in the life expectancy of diabetic individuals. This increase is expected to cause a rise in the burden of DR since most of these patients may not be able to receive a timely eye examination or treatment for diabetes [9]. Furthermore, due to the risk of DR, people with diabetes are 25 times more likely to become blind than non-diabetics [10]. In 10 years, 71–90% of type 1 diabetics will have developed DR, with the incidence rising to 95% in 20–30 years, while the incidence in type 2 diabetics is 67% after ten years [11]. This DR may be complicated due to severe diabetic macular oedema and proliferative DR leading to visual impairment [11]. DR and the resulting blindness are expensive to treat, thus placing an economic burden on the patient and their family and significantly impacting the world’s health systems [12]. Early screening and diagnosis for DR are essential since most patients have no symptoms until visual impairment, and it is less costly compared to the cost and effects of managing blindness as a disability [11]. Screening can help prevent blindness among 90% of people with diabetes. However, this is yet to be achieved in Uganda. This is because the gold standard diagnostic tools like the ophthalmic fundus camera or an Indirect ophthalmoscope are non-portable, inaccessible and expensive for hospitals or clinics in Uganda [13].

LMICs need a unique modality that combines affordability, availability and a short learning curve to screen for DR. Furthermore, they need training and expertise, limiting their use to the ophthalmologists who are only 40 in Uganda. Therefore, many diabetic patients may not access proper fundus examination for DR despite the adequate numbers of ophthalmic clinical officers in the peripheral health centres. The smartphone-based camera can bridge this gap in LIMCs; however, there is no published data to support its use in our setting.

The International Council of Ophthalmology (ICO) and Uganda Ministry of Health guidelines recommend screening, follow-up, referral, and early diagnosis and treatment of DR [14–16]. This is in line with the third sustainable development goal (SDG) that ensures good health and well-being for all [17, 18].

In the last decade, there has been a trend to develop cost-effective, portable, and easy-to-use devices by minimally trained personnel [11, 19, 20]. These devices integrate smartphones with a lens or an adapter that can allow visualisation of the retina. This has the potential to revolutionise eye care, especially in low-income countries. Among the many products, the Portable Eye Examination Kit (PEEK) retina’s use in the assessment of retinal pathology has been documented by several studies worldwide [19, 21–23]. The PEEK retina is a smartphone-based adapter attached to the back of the phone. Using downloaded software on the smartphone allows high-resolution fundus images to be obtained quickly and stored or distributed remotely for grading and second opinions in case of diagnostic doubt [24, 25]. The above qualities make the PEEK retina easy to use compared to the traditional portable direct and indirect ophthalmoscopes and fundus cameras that are bulky and require specialist skills, thus highly limiting their use by untrained personnel [26]. In Africa, it has been used to diagnose malaria retinopathy in Malawi [27] and to image the optic disc in Kenya [28]. However, there is limited data on its use as a screening or diagnostic tool for diabetic retinopathy.

Therefore, this study aimed to determine the sensitivity and specificity of the smartphone-based device (PEEK retina) compared to the standard tabletop fundus camera (Zeiss Visucam 200) as the gold standard for diagnosing diabetic retinopathy. We also tested the reliability of the Peek retina by grading the diabetic retinopathy and comparing it with the Zeiss Visucam 200.

## Materials and methods

### Study design and setting

This descriptive hospital-based cross-sectional study was conducted in the Diabetic Clinic of the Department of Medicine, Kiruddu National Referral Hospital, between January and March 2020. The hospital is located in Kampala, Uganda and serves as the primary inpatient and outpatient hospital for Kampala and its surrounding districts. The Diabetes clinic operates weekly and reviews approximately 80 to 110 patients daily. It is run by one ophthalmologist and five ophthalmic clinical officers. The clinic equipment used to examine the posterior segment of the eye includes a slit lamp, 90D and78D lenses, a direct ophthalmoscope and an indirect ophthalmoscope.

### Study participants and data collection

All known diabetic patients (Type 1 or Type 2) previously diagnosed and taking medicine from the diabetes clinic who were 18 years and above, had given written informed consent and were willing to undergo the study procedure were recruited. We excluded participants with the following: a mental disorder, contraindication to mydriasis, allergy to tropicamide eye drops, hazy optical media and those too sick to undergo the study procedure.

### Study procedure and data collection

We collected data from the patient’s medical records card using a standardised pretested questionnaire after the patient had been reviewed by the physician in the diabetes clinic that day. This included the demographics, clinical presentation, diagnosis, duration and family history of diabetes mellitus, fasting blood glucose measurement, blood pressure measurements of the hypertensive patients, and history of kidney and sickle diseases. We measured the participants’ visual acuity using a Snellen’s chart and examined them with portable slit-lamp biomicroscopy. Findings of the anterior segment were recorded for each patient. Tonometry was done using a handheld ICARE tonometer (Icare® TA01i, Tiolat Oy, Helsinki, Finland), and Visual fields were tested by the confrontational method. Eye movements were tested in all gazes seeking any paresis or paralysis of extraocular muscles. Each patient then underwent mydriatic dilation with Tropicamide 1% eye drops.

### Fundus examination

The PEEK is a comprehensive and integrated smartphone-based tool kit that contains a set of core tests needed for eye screening. It is designed for use by practitioners with minimal to no training. It comprises a smartphone application and a low-cost adapter for retinal imaging. Both are optimised for ease of use, and it allows operators to test for the core vision problems that are visual acuity, colour, and contrast sensitivity, image grading of cataracts and photos of the back of the eye to be taken, saved, and sent to experts for diagnosis, follow-up, and arranging treatment.

### PEEK retina

The PEEK retina is a plug-in imaging system with adjustable light and power to illuminate the retina. Users can change the amount of light to illuminate the retina by choosing one level out of three. Since it has its light source, Peek Retina has a universal clip to attach to any smartphone. It also has an Android application named Peek Retina Camera that enables it to capture photos and record videos. However, any camera application with iPhone and Windows Phone models can be used to capture images by adjusting manual settings in terms of autofocusing, clarity, and brightness.

It also has a synthetic eye model box for synthetic data capture, thus providing a package with eight different retina images with different eye disease conditions ranging from the normal eye to proliferative diabetic retinopathy. The merits of the PEEK retina include its portability, ease of use with a short learning curve, cost-effectiveness, availability, and ability to make a remote diagnosis [29, 30].

With the eye fully dilated, each participant’s fundus image was recorded as a video by the PEEK retina using a Samsung S8 edge plus(model SM-G955FD) mobile digital camera(Samsung C&T Corp., Seoul, and the Republic of Korea). The peek retina adapter was attached to the phone’s native camera, aligned and tightened with a knob to hold the device in the correct position. Since the PEEK retina has its light source, the smartphone flashlight was disabled, providing the Peek retina software with a retinal imaging function.

The participant was given a target, and the video was acquired from the central posterior 45 degrees of the retina. The video length depended on the participant’s cooperation and the ability to acquire the required images from the posterior pole. After that, the participant’s fundus picture of the posterior pole was then taken by the ZEISS VISUCAM 200 ophthalmic fundus camera(Carl Zeiss Meditec AG, Jena, Germany). The principal investigator reviewed the videos and pictures from both devices to diagnose DR and graded them using ICDRDS classification [31]. If the optic disc was not evident in the PEEK video or the image of the Zeiss Visucam 200, it was classified as an image not clear.

### Sample size

Using Bruderer’s sample size estimation formula for determining sensitivity and specificity [32], a sample size of 286 participants (572 eyes) was obtained.

### Sampling procedure

We used systematic random sampling with a sampling interval, k = 4, which was obtained by dividing the sampling frame size by the study sample size. According to the time of arrival at the clinic, every fourth patient was enrolled in the study. If they declined to join the study, the subsequent fourth patients’ in the series were selected. The first patient was picked from the first four patients using random number tables, and all patients were seen after being reviewed by the physician.

### Statistical analysis

For descriptive statistics, continuous variables were summarised as mean and standard deviation if normally distributed or median if not normally distributed. The categorical variables were summarised into frequencies and percentages. Participants whose images were recorded as unclear were not included in the calculation for sensitivity and specificity. Considering the Zeiss Visucam 200 as the gold standard, sensitivity and its 95% Confidence interval were determined by getting the number of diabetic patients diagnosed with DR by the smartphone-based retinal photography (PEEK-retina) divided by all those that were diagnosed as positive by the gold standard. Specificity and its 95% Confidence interval were determined by getting the number of diabetic patients who were correctly diagnosed as not having diabetic retinopathy by PEEK retina divided by all those diagnosed as negative by the ZEISS VISUCAM 200. We determined the reliability and the concordance of the PEEK retina and ZEISS VISUCAM 200 ophthalmic fundus camera in DR staging. The proportion of concordant and discordant pairs was obtained as a percentage of all the pairs of observations. All analyses were done in STATA version 14.0.

### Ethical considerations

We obtained ethical approval from the School of Medicine Research and Ethics Committee (SOMREC) of Makerere University and granted an approval number of # REC REF 2020–011. We also sought voluntary written informed consent from the participants before they joined the study.

## Results

**Fig 1:**
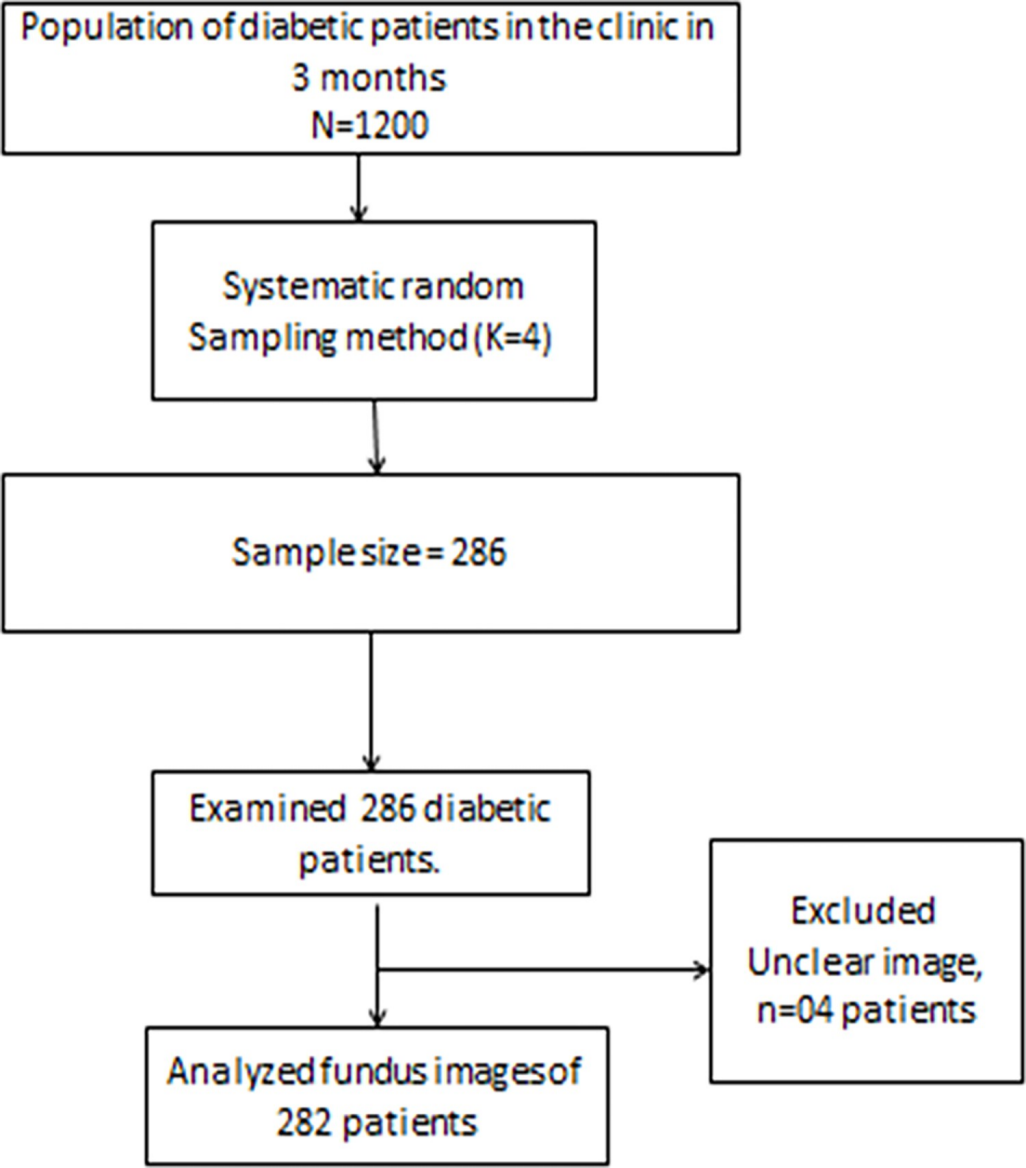
Diabetic patient’s flow chart in Kiruddu National Referral Hospital from January to March 2020.

We recruited 286 participants into the study who had a mean age of 51 and a standard deviation of ± 11 years. Most study participants were female, 213/286 (74.5%). More than half of the participants had primary education, 168/286 (58.8%) and 93.4% (267/286) of the participants had type 2 DM. We excluded unclear images from four patients during the result analysis, as shown in " Fig 1".

From "Table 1", The majority (93.4%) of the participants had type 2 diabetes, and more than half had hypertension. A few participants had sickle cell disease (3), while most had a positive family history of diabetes.

**Table 1:** Baseline characteristics of the diabetic patients in Kiruddu National Referral Hospital from January to March 2020.

Characteristics	All (N = 286), %	Female (N = 213), %	Male (N = 73), %	P value
**Diabetes type**				
1. Type 1 DM	19 (6.6)	10 (4.7)	9 (12.3)	0.024
2. Type 2 DM	267 (93.4)	203 (95.3)	64 (87.7)	
**Age of participants (median (IQR))**	51(44–58)	50 (43–56)	54 (47–60)	0.016
**Education level**				
Primary	168 (58.8)	124 (58.2)	44 (60.3)	0.117
Secondary	93 (32.5)	74 (34.7)	19 (26.0)	
Tertiary	23 (8.0)	13 (6.1)	10 (13.7)	
None	2 (0.7)	2 (0.9)	0	
**Hypertension Categories**				
No hypertension	89 (31.1)	63 (29.6)	26 (35.6)	0.302
Grade 2:140-159/90-99	73 (25.5)	54 (25.34)	19 (26.0)	
Grade 3:> = 160/100	115 (40.2)	91 (42.7)	24 (32.9)	
Missing	9 (3.2)	5 (2.3)	4 (5.5)	
**Medical History of Hypertension**				
No	128 (44.8)	95 (44.6)	33 (45.2)	0.929
Yes	158 (55.2)	118 (55.4)	40 (54.8)	
**Medical History of Sickle Cell Disease**				
No	283 (99.0)	210 (98.6)	73 (100.0)	0.308
Yes	3 (1.0)	3 (1.4)	0 (0.0)	
**Medical history of renal disease**				
No	284 (99.3)	213 (100.0)	71 (97.3)	0.15
Yes	2 (0.7)	0 (0.0)	2 (2.7)	
**Medical History of DM**				
No	117 (40.9)	85 (39.9)	32 (43.8)	0.556
Yes	169 (59.1)	128 (60.1)	41 (56.2)	

P-values by t-test for continuous variables and Chi2 test for binary/categorical variables.

In "Table 2", PEEK retina correctly identified 84% (95% CI: 70.9–83.5) of the patients with DR and 79.9% (95% CI: 76–83.5) of those who indeed did not have DR. Out of the patients identified as having DR by PEEK retina, 30.9% (95% CI: 23.2–39.4) were true positives. Of the patients identified as not having DR by PEEK retina, 97.9% (95% CI: 95.9–99.1) were True Negatives. The positive likelihood ratio reflected a slight increase (4.18) in the likelihood of having DR. In contrast; the negative likelihood ratio showed a 0.2 increase in the likelihood of having DR. Fifty-four images were recorded as unclear because, therefore, they were not considered in the calculation of sensitivity, and specificity. These were also not graded in the calculation of the agreement.

**Table 2:** Sensitivity and specificity to detect any DR in both rights and left eyes in diabetic patients attending Kirrudu National Referral Hospital from January to March 2020.

Peek Retina	Gold standard						
	Negative	Positive	Total		% (95% CI)		% (95% CI)
**Negative**	374	8	382	**Specificity**	374/468, 79.9 (76–83.5)	**NPV**	97.9 (95.9–99.1)
**Positive**	94	42	136	**Sensitivity**	42/50, 84.0 (70.9–92.8)	**PPV**	30.9 (23.2–39.4)
**Total**	468	50	518				
**Likelihood ratio (+)**		4.18 (3.36–5.2)					
**Likelihood ratio (-)**		0.2 (0.11–0.38)					

Overall the agreement in "Table 3" between PEEK Retina and Zeiss Visucam 200 was high, i.e., the agreement for mild NPDR and severe NPDR was 94.06% and 91.3%, respectively for the right eye and 93.0% and 96.9% for the left eye.

**Table 3:** Agreement between PEEK retina and the Zeiss Visucam 200 in the staging of DR in diabetic patients attending Kirrudu National Referral Hospital from January to March 2020.

Agreement between the PEEK Retina and Zeiss Visucam in Grading DR in the Right eye						
Peek Retina	Gold Standard		Agreement, %	Expected agreement,%	Cohen’s Kappa	P-value
	**Mild NPDR**					
**Mild NPDR**	**No**	**Yes**	94.06	94.1	-0.01	0.601
No	269	16				
Yes	1	0				
	**Moderate NPDR**					
**Moderate NPDR**	**No**	**Yes**	88.81	79.8	0.45	<0.001
No	238	28				
Yes	4	16				
	**No Apparent Retinopathy**					
**No Apparent Retinopathy**	**No**	**Yes**	77.62	63.6	0.39	<0.001
No	35	17				
Yes	47	187				
	**PDR**					
**PDR**	**No**	**Yes**	98.60	98.6	-0.01	0.541
No	282	3				
Yes	1	0				
	**Severe NPDR**					
**Severe NPDR**	**No**	**Yes**	91.3	90.2	0.11	<0.011
No	259	4				
Yes	21	2				
**Agreement between PEEK Retina and Zeiss Visucam 200 in Grading DR in Left eye**						
**Left eye**			**Agreement,%**	**Expected agreement,%**	**Cohen’s Kappa**	**p-value**
	**Mild NPDR**					
**Mild NPDR**	**No**	**Yes**	93.0	92.4	0.04	0.031
No	265	18				
Yes	2	1				
	**Moderate NPDR**		85.3	83.2	0.13	0.008
**Moderate NPDR**	**No**	**Yes**				
No	239	32				
Yes	10	5				
	**No Apparent Retinopathy**		79.0	63.0	0.43	<0.001
**No Apparent Retinopathy**	**No**	**Yes**				
No	38	12				
Yes	48	188				
	**PDR**		99.0	97.6	0.57	<0.001
**PDR**	**No**	**Yes**				
No	281	2				
Yes	1	2				
	**Severe NPDR**					
**Severe NPDR**	**No**	**Yes**	96.9	96.2	0.18	<0.001
No	276	9				
Yes	0	1				

## Discussion

In this hospital-based cross-sectional study, we tested the validity of the Portable Eye Examination Kit (PEEK) retina for the diagnosis of DR in the Kiruddu National referral hospital diabetes clinic. We compared it to a standard ophthalmic fundus camera (Zeiss Visucam 200). The PEEK retina uses a low-cost adapter attached to the phone camera. Peek retina software downloaded on the smartphone provides a small-sized, portable, low-power, and affordable biomedical imaging device with a retinal imaging function [29]. This can be used to perform fast DR screening with less expertise than retinal imaging with a fundus camera which is a time-consuming manual process that needs the expertise to capture retinal images.

The PEEK retina had an overall high sensitivity (84.0%) and specificity (79.9%) compared to the Zeiss Visucam 200. Both devices had a high agreement in detecting the different grades of DR. The rate of recorded ungradable (unclear) images was acceptably low, approximately 9% and these were excluded from the above calculations.

Our participants had a mean age of 51 years, which is no different from other studies [9, 33]. This is because most (93.4%) participants presented with Type 2 diabetes, mainly affecting persons aged 45 years and above [34]. The majority of our participants were females, and this could have resulted from the good health-seeking behaviour of females compared to their male counterparts, especially between the age of 10 and 60 years, which is the age range of most of our participants, and this is no different from other studies globally [35, 36].

Sensitivity and specificity are the main assessment factors to validate a screening tool or device. Since sensitivity is the actual positive rate of a device, this means that the PEEK retina would approximately correctly identify 84 out of 100 participants that indeed had DR as having DR. This value is very high; hence the device can be used as a screening tool for DR since it would not misclassify most of the participants as not having DR yet they do. The findings of our study are similar to those of other studies done in Africa and elsewhere in the world regarding smartphone-based retina photography [20]. Ramachandran in India found the sensitivity to be 92.7%, while Sengupta et al. recorded 93.1% sensitivity [9, 37]. This similarity in the degree of sensitivity could have been because of the excellent image quality of both devices. The slight difference in sensitivity could have been because of the difference in the study population. That is to say, our study was on blacks, and the majority were females who tend to fear investigative procedures, while the studies by Ramachandran & Sengupta et al. were mainly on male Indians (64.5%) and (65%), respectively [9, 37].

Findings in our study showed a specificity of 79.9% for the PEEK retina; this means that the PEEK retina would approximately correctly identify 80 out of 100 participants without DR as not having DR. This value is high, which means the device is excellent as a diagnostic device for DR because of the few false negatives thus, reducing misclassification bias. This result is consistent with the other smartphone-based fundus camera studies from India that reported 98.4%, 94.9% and 94.3% [9, 20, 29, 37].

In our study, the PEEK retina also had an NPV of 97.9%, while the PPV was 30.9%. Therefore, the probability of a positive test for DR to be positive is 0.309, which is low for determining positive tests. However, the prevalence affects the above two parameters, which can explain the difference from other smartphone-based fundus camera studies [9, 37–41]. In addition, the high NPV results are comparable to a USA study that showed its accuracy of detecting healthy retina (No apparent DR) being higher than that for detecting DR in Peek Retina images [29].

The agreement of PEEK retina in the diagnosis of Diabetic Retinopathy had most percentage agreements more significant than 90% for the right eye and the left eye in severe NPDR and PDR; however, the agreements for no apparent DR were below 80%. Similarly, an agreement of 0.91 was reported by Toy et al. [20] after examining 100 eyes of 50 patients at Byers Eye Institute, Stanford University, USA, where the same gold standard was used to evaluate the reliably of PEEK retina as a cheap alternative for the screening of DR. This implies that PEEK retina will give consistent results 90% of the time. These values are high and not different from those of other smartphone-based fundus photography studies [9, 37], thus making the PEEK retina a reliable tool for screening all grades of DR. The percentage agreement values for mild and moderate NPDR are not as high as those for PDR (98.6% for the right eye and 99% for the left eye) and are comparable to other studies where Sensitivities for mild NPDR and moderate NPDR were 57.1% and 42.9%, respectively. In comparison, that of PDR was 80% [42]. This could be because the PEEK retina has a small field of view. A study to determine the performance of smartphone-based fundus imaging system in detecting DR found that this performance decreases as the field of view of the smartphone-based retinal systems get smaller, where iNview is the largest and iExaminer is the smallest. The accuracy of the different smartphone systems was 61%, 62%, 69%, and 75% for the i-examiner, D-Eye, Peek Retina, and iNview images, respectively [29]. Secondly, there were flashes in the pictures, which could have deterred the grading of the early changes in DR since some changes are subtle and need to be critically analysed compared to the more obvious advanced retinal changes. Since Peek Retina captures smaller areas of the retina and mainly their images are focused on the optic disk and its surroundings, lesions that generally appear in the peripheral retina might be left out. Furthermore, our study was conducted in a diabetes clinic where patients with diabetes were referred for DR screening; therefore, the different grades of DR were not evenly distributed in the study population. However, other SPBFI studies in the grading of DR sampled carefully curated study populations to include almost equal numbers of eyes with different levels of DR, ranging from no apparent DR to PDR [9].

In this study, video recording was used for the PEEK retina instead of photography because of the small field of view that the peek retina gives. Smartphone-based fundus video recording has also been used to extract images in a study done in retinopathy of prematurity with an outstanding image quality of 98%; however, no studies show its use in screening DR [43]. Furthermore, a study done to compare the four most used smartphone-based fundoscopies reported their radius of printed fundus images as 32%, 40%, 45%, and 94% for iExaminer, D-Eye, Peek Retina, and iNview, respectively [29]. Therefore the PEEK retina fundus video enabled us to record a wider field since it is also essential to assess the peripheral retina when screening for DR.

The strengths of this study included the following: The study used the gold standard to reliably evaluate the PEEK retina as a cheap alternative for the screening of DR. Optic disc assessment was easier with the PEEK retina, and this was also demonstrated in other studies [28]. This could be because optic disc assessment does not require a well-dilated pupil or a cooperative patient.

When using the PEEK retina in this study, several limitations were encountered. Firstly, this study was hospital-based; therefore, results from this study can only be generalised to tertiary health centres in resource-limited settings with diabetic patients but not the entire population. Secondly, there were white artefacts at the images’ edge, which led to missing some signs like microaneurysms. This could be because the Peek Retina imaging system includes light to illuminate the retina, and a smartphone captures the image; therefore, it does not reflect the flashlight. In addition, this device could not be used on an undilated pupil and had a limited field of view. This was minimised by asking the patient to look into different fields of gaze to get a periphery image and record videos from which the images were captured. Furthermore, the device also needed good cooperation from the patient to be able to obtain interpretable images.

Thirdly, using a single mobile model may have also affected the image quality. In this study, the Samsung model was used, while other studies have used the i phone and HTC phones [43]. In addition, there is no available data comparing the different smartphone models; however, smartphones generally have a lower image quality than the fundus camera due to their fewer controllable parameters and lenses [29].

## Conclusion

PEEK retina has very high sensitivity and specificity, making it suitable for screening DR in a resource-limited setting. It can reliably diagnose no apparent DR properly most of the time. That being the majority of the population, PEEK retina helps by saving them the stress and money to undergo gold standard ophthalmoscopy, which is expensive and not easily accessible.

We, therefore, recommend the PEEK retina to be used in the screening of diabetic patients for DR, especially in lower health units with poor infrastructure, given its high overall sensitivity and specificity, low cost, portability, and ease of use, cloud storage and the availability of smartphones. These advantages of a PEEK retina smartphone-based system can be harnessed to improve telescreens’ penetration of DR, especially in resource-poor settings of the developing world. In addition, more research is needed in the application of the PEEK retina while using the different types of smartphones to obtain quality retina images and to incorporate smartphone-based systems that offer a way to analyse and evaluate eye disease using the PEEK retina by using image processing techniques for grading retinal images, especially in resource-limited settings.

## Supporting information

S1 Fig (TIF)

S1 Appendix (DOCX)
