# Supplementary figures and images for: Validity of smartphone-based retinal photography (PEEK-retina) compared to the standard ophthalmic fundus camera in diagnosing diabetic retinopathy in Uganda: A cross-sectional study
Source: PLoS One. 2022 Sep 6;17(9):e0273633. doi: 10.1371/journal.pone.0273633 (PMC9447889; doi:10.1371/journal.pone.0273633)

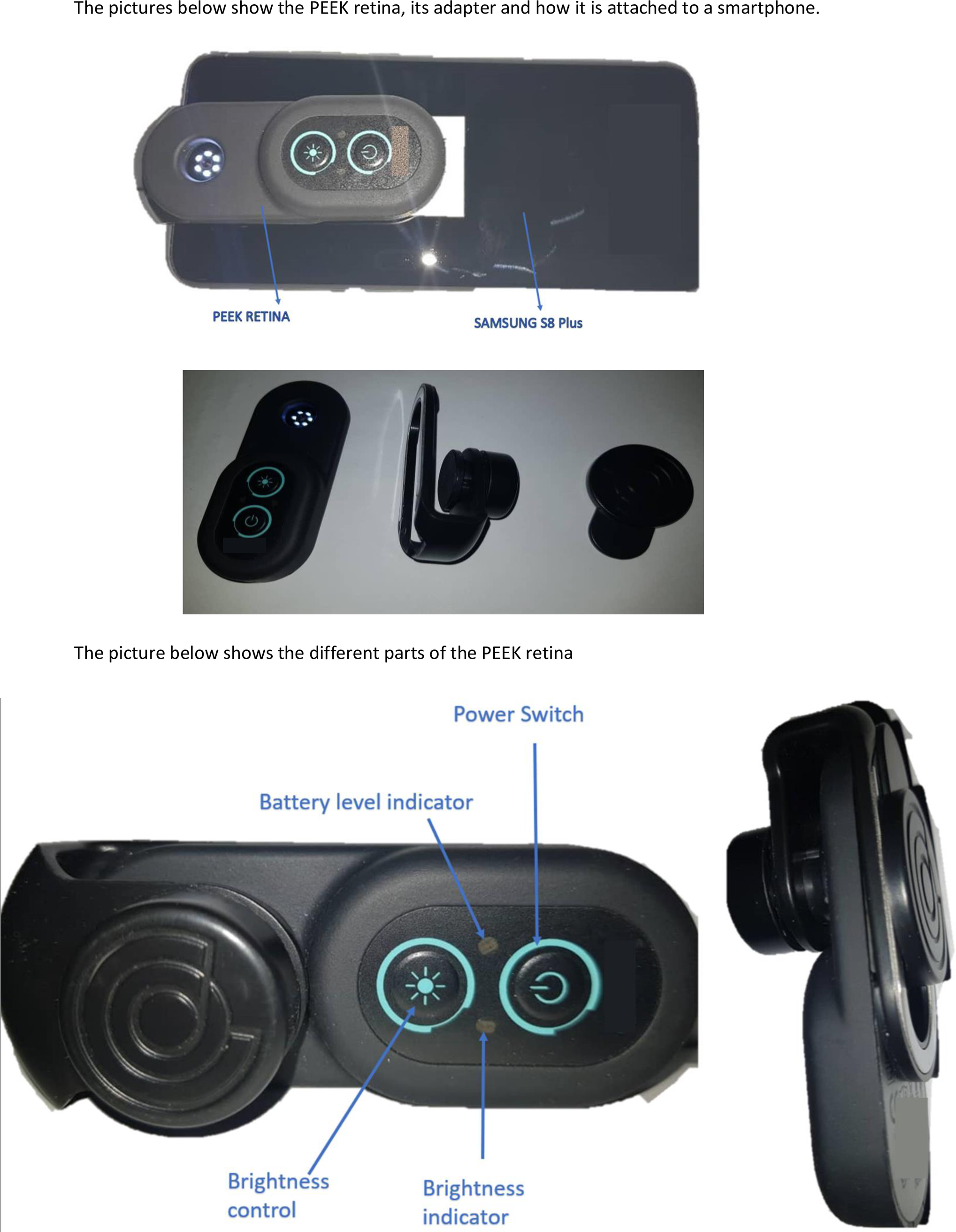

Supplement: S1 Fig — (TIF) [file pone.0273633.s001.tif]
