# Supplementary material for: Validity of smartphone-based retinal photography (PEEK-retina) compared to the standard ophthalmic fundus camera in diagnosing diabetic retinopathy in Uganda: A cross-sectional study
Source: PLoS One. 2022 Sep 6;17(9):e0273633. doi: 10.1371/journal.pone.0273633 (PMC9447889; doi:10.1371/journal.pone.0273633)
Supplement: S1 Appendix — (DOCX) [file pone.0273633.s002.docx]

|  | **Testing Device** | **Gold Standard** |
| --- | --- | --- |
| Device | PEEK Retina adapter with Samsung Galaxy S8+ | Zeiss VISUCAM 200 |
| Field of view | 20°to 30°[1] | 45° and 30° |
| Pupil Diameter | Must be dilated | ≥ 4.0 mm  ≥ 3.3 mm (30° small pupil mode) |
| Capture sensor | Rear camera of the S8+ Dual Pixel 12.0 MP | CCD 5.0 mega pixels |
| Product Weight | PEEK retina adapter 35 and S8+ is 173g | 30 kg |
| Portability | Portable | Non-Portable |
